# Design and Performance Assessment of a Solid-State Microcooler for Thermal Neuromodulation

**DOI:** 10.3390/mi9020047

**Published:** 2018-01-27

**Authors:** José Fernandes, Estelle Vendramini, Ana M. Miranda, Cristiana Silva, Hugo Dinis, Veronique Coizet, Olivier David, Paulo Mateus Mendes

**Affiliations:** 1 CMEMS, University of Minho, 4800-058 Guimarães, Portugal; fernandes.jmn@gmail.com (J.F.); aana.mmargarida@gmail.com (A.M.M.); a72347@alunos.uminho.pt (C.S.); hugodcdinis@gmail.com (H.D.); 2 Grenoble Institut des Neurosciences, U1216 Inserm, Université Grenoble Alpes, 38400 Grenoble, France; estelle.vendramini@sfr.fr (E.V.); veronique.coizet@univ-grenoble-alpes.fr (V.C.); Olivier.David@inserm.fr (O.D.)

**Keywords:** solid-state cooling, microsystem integration, thermal simulation, thermal neuromodulation, microdevice packaging, biomedical microdevice, neuronal, implantable

## Abstract

It is well known that neural activity can be modulated using a cooling device. The applications of this technique range from the treatment of medication-resistant cerebral diseases to brain functional mapping. Despite the potential benefits of such technique, its use has been limited due to the lack of suitable thermal modulators. This paper presents the design and validation of a solid-state cooler that was able to modulate the neural activity of rodents without the use of large and unpractical water pipes. A miniaturized thermal control solution based exclusively on solid-state devices was designed, occupying only 5 mm × 5 mm × 3 mm, and featuring the potential for wireless power and communications. The cold side of the device was cooled to 26 °C, while the hot side was kept below 43 °C. This range of temperatures is compatible with brain cooling and efficient enough for achieving some control of neural activity.

## 1. Introduction

Recent advances in technology have been paving the way to new medical integrated solutions for the treatment, diagnosis, prevention, and even for the replacement of human body functions. Such technological advances, like miniaturization and low power consumption, took the research areas to a new level where microdevices are now capable of reaching so-far inaccessible applications. Fully-implantable microsystems are becoming a reality and their growing market is expected to reach more than $54 billion in value, until 2025, in developed countries [1]. These implantable devices could have hundreds of different usages, such as the diagnosis or treatment of various neurological disorders, cardiovascular problems, and ophthalmologic diseases [2].

The nervous system can greatly benefit from this technological progress, where microdevices should be able to measure and actuate on single neurons, giving new information and treating places that were unreachable before [3,4]. One possible approach, called neuromodulation, consists of the control of neural cells’ behavior, and includes every approach that changes the neurons’ biochemical process or electric signals. This is achieved by suppressing or facilitating the flow of action potentials to accomplish a specific brain function.

In this context, thermal neuromodulation may play a significant role. An earlier work from Baldwin and Frost (1956) [5] reported that cooling the brain to 30 °C or below was effective at suppressing electrical discharges on brain cells, and could stop strong electrical signals, such as seizures. Many other studies followed; for example, thermal regulation resorting to cooling, also known as hypothermia, was tested to treat insomnia [6], and is also used on a more regular basis to prevent brain injuries and traumas after strokes [7], or on newborns to prevent serious brain injuries [8]. Overall, epileptic seizure suppression was the main application of brain cooling and will be the main focus of this work.

Epilepsy affects more than 50 million people worldwide, and 30% to 40% of patients are not responsive to antiepileptic medication [9]. Thus, thermal neuromodulation presents itself as an alternative solution for this group of patients. Reducing the electrical signals before the seizure intensifies can be a solution to suppress the latter. This method is already used on site when performing brain surgeries: a cold liquid (0 °C) is poured on the brain’s surface to suppress epileptic seizures that can randomly start. Cooling the brain is, therefore, one of the quickest and most effective responses available to quickly stop a seizure. Consequently, developing a small device capable of detecting and suppressing electric signals that can be implanted on the brain is a technological solution of interest for the chronic control of drug-resistant focal seizures.

To suppress seizures, a temperature of around 20–30 °C or less is required [5,10]. This way, the device must be able to reduce the tissue’s temperature by at least 7–17 °C, considering that the brain’s temperature is around 37 °C. In order to be safe, this device has also to maintain temperatures within safety limits of biological tissues, which implies that its hot side cannot surpass 43 °C and the cold side must not reach sub-zero temperatures [11,12]. Going out of this temperature range can lead to irreversible thermal damage. Additionally, cooling for long periods of time can reduce the motor function of the whole body and, in extreme cases, it can also cause pneumonia [13].

In the literature, small systems for thermal modulation in the brain have been reported to produce good results. Rothman and his team were some of the first authors proposing the use of focal cooling to terminate seizures, and have published the evolution of their work based on different devices. However, all of them required water to cool down the system [14,15,16]. In a different kind of device, Ahiska made a complete model of a thermoelectric helmet that can cool down the brain in a non-invasive way. Nevertheless, and much like the previous device, water was required to cool down the hot side of the Peltier modules [17]. In his review, Fisher presented a way of cooling the brain with only a small implanted Peltier. However, since no proper heatsink was used to cool down the thermoelectric module, after turning off the Peltier, the heat from the hot side would dissipate in the rat’s head, damaging its cells [18]. In 2011, Hou and his research group developed an integrated wireless system to cool down and stop seizures in the brain. This system required no batteries and was powered resorting to wireless power transfer. The paper presents good results in terms of communication between the device and the external source of power, but it is difficult to understand the accomplished brain cooling results. One main conclusion that can be made is that the wirelessly-transmitted power was not enough to stop seizures [19]. Another example comes from Fujji and his fellow researchers, who have developed one of the most advanced systems for brain cooling. Their approach was based on the use of a small Peltier together with water cooling to achieve the desired tissue temperature. Such a device was able to cool the brain tissue down to 10 °C without causing any damage to the cells. Consequently, seizures below the focal points were completely terminated. The system was tested and placed in rats’ brain cortex [20]. Furthermore, there are also examples of devices that do not use Peltier modules to reduce the temperature. Cooke and his research group used PDMS tubes to circulate cold water and cool down the cells. In their research, two types of devices were developed: one to place on the surface of the brain and another to place in the brain grooves (also known as sulcus) [21]. However, these studies were based only on the effectiveness of the focal cooling and did not target cheap and small devices that can be used in vivo, as they had large cooling modules with considerable areas that were not portable and were impossible to implant in the brain.

To sum up, thermal neuromodulation, despite its reported benefits, is not used as much as expected, mainly because the available cooling devices are, in general, too large for practical use, even for research purposes [22]. Such devices are far from being suited for permanent use in humans, or for testing with monkeys or rodents. This paper, thus, presents a solution to obtain a solid-state microcooler that is potentially better suited for its practical use. While developing this solution, the power requirements and volume constraints needed to obtain a reliable, portable, and fully-implantable device were considered, in order to be used on a rat’s brain. Simulations and measurements on phantoms and rats were performed to validate this effective, small, and energy-efficient device.

## 2. Cooling Device Design

**Figure 1:**
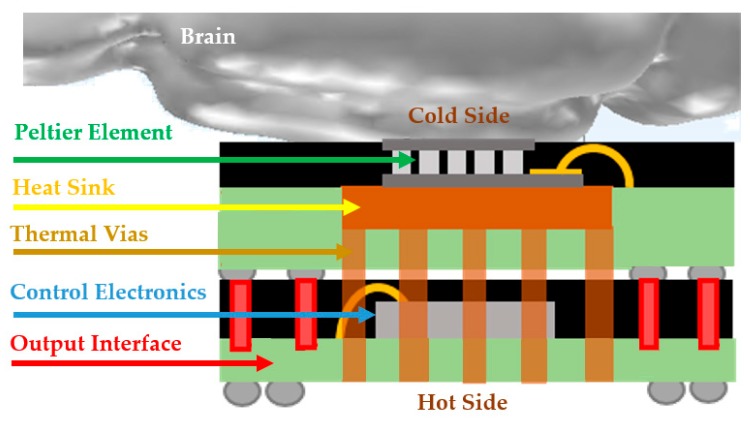
Developed miniaturized thermal neuromodulator. The main elements are described along with the figure. The Peltier element is used to obtain a cold volume; the heat sink, together with the thermal vias, will remove the heat away from the cold side; the control electronics is a chip used to generate the signals to operate the Peltier cooler; the output interface is set on interconnections that allows to interconnect the device with other subsystems, like a battery or an antenna. The non-highlighted aspects are non-relevant interconnections between sub-systems.

A mechanism to move heat from the defined neuronal region to somewhere else in the brain is required to achieve the desired local temperature in the range of 20–30 °C [5,10]. The proposed solution to obtain a solid state microcooler is shown in Figure 1.

The design of such device must take into account three aspects. Firstly, some other volume (e.g., water, or even brain tissue) will suffer an increase in temperature, as thermal energy is moved from one location to the other. Secondly, the heat to be transferred will increase proportionally with the cooled volume. Finally, the electric energy required by the “heat pump” will increase proportionally with the amount of heat to be transferred. Thus, the volume to cool down must be precisely defined, in order to avoid spending unnecessary energy and to prevent dealing with too much heat. Additionally, the more efficient the heat transfer mechanism is, the less energy is required to operate the device, which is very important since such devices are usually battery-operated.

### 2.1. Cooling Mechanism

The first step of a thermal modulator design is the selection of the cooling mechanism. Several methods are available [23], each of them with different performances regarding energy use. In this work, only solid-state-based solutions that operate in the body temperature range are of interest. These solutions can be based on the following effects: thermionic, magnetocaloric, thermoelastic (elastocaloric), electrocaloric, and thermoelectric.

The thermionic cooling effect is observed when energetic hot electrons leave a cathode surface. This mechanism takes energy away from the cathode, which leads to cathode cooling [24]. Although this method is very efficient, it has low thermal cooling power at ambient temperatures [25,26,27], which renders it ineffective for the scope of this work. The magnetocaloric effect resorts to a magnetic field to obtain a cooling effect [28,29]. This is a very interesting effect since it allows operating the cooling device wirelessly. Nevertheless, a strong magnetic field is required to operate the device, which either draws a large current or requires a large volume, if a permanent magnet is used. Another available option would be the electrocaloric effect, where an applied electric field produces a temperature change [30,31]. One drawback of the solution is the need to use hundreds of MV/m to operate the device, which may be difficult to obtain with standard electronics in an integrated solution, thus making this solution unpractical. Another promising technique is the elastocaloric effect, where a temperature change is produced in response to a mechanical stress [32,33,34,35,36,37]. However, alongside with its very early stage of development, the use of such a technique in a tiny device would require the use of MEMS devices to apply the required stress, or the use of piezoelectric elements [38], which adds complexity, and potentially extra volume to the device.

Finally, another option is to implement a device based on the thermoelectric effect, such as the Peltier effect, where an electric current may be used to produce a temperature gradient [39,40]. This is not the best solution in terms of efficiency [23] but, from the available solutions, it is the most developed, with many commercially-available devices, and with an excitation mechanism (an electric current) that can be easily generated by standard electronics. This was the approach we propose to build a functional device for testing purposes (as shown in Figure 1).

### 2.2. Peltier Modeling

Since a device to be implanted inside a head should, desirably, be portable and rely on batteries, its power consumption must be carefully managed and, ideally, minimized. When the device is particularly power-consuming, much like a Peltier device is, this becomes an especially critical design step. After selecting the thermoelectric effect as a solution to obtain the required cooling of neural tissue, it was thus necessary to understand how much electrical power would be required to reach the desired temperature and what was the consequent device size. Additionally, it was necessary to understand if the heat generated was manageable by a reasonably-sized passive heatsink. To have an effective operation, the cold side temperature must reach around 20 °C, keeping in mind that the hot side must not go above 43 °C [12]. To comply with such specifications, simulations were used to predict the required current to operate the device and to design the heatsink, a fundamental element in the device.

**Figure 2:**
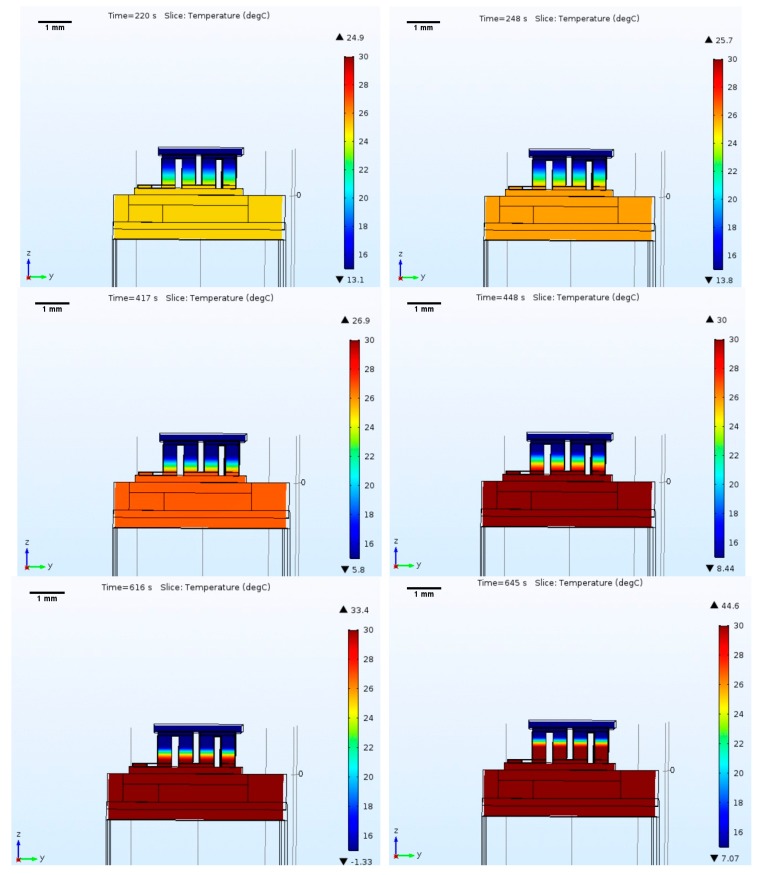
Simulation results with Peltier and heatsink operating in free space. The figure shows the temperature along the Peltier element, as well as the heatsink region nearby, which will be the hottest area. In addition to the color map showing the temperature distribution, also shown is the ▼-lower, and ▲-higher temperatures recorded in the structure for the selected time stamps (**top**—100 mA; **middle**—200 mA; **bottom**—400 mA; **left**—minimum temperature achieved; **right**—temperature stabilization).

The thermal modulator operation was evaluated using a model built and simulated using the thermoelectric equations, and the heat-transfer in solids and electric currents models in COMSOL (COMSOL Group, Stockholm, Sweden). The first test aimed to analyze the device operation in air, at ambient temperature, where the heat sink was placed on the hot side of the Peltier (custom thermoelectric 00801-9U30-10RU3). On the surfaces of the thermoelectric device and heat sink, an external natural heat convection to 24 °C (ambient temperature) was applied (on the pillars, the temperature was isolated). The initial temperature was also defined at 24 °C, simulating the temperature of the laboratory where the Peltier will be tested. A transient analysis was performed, where a square wave was applied on the input pad, turning on the Peltier. Figure 2 shows the implemented model and the obtained results.

The heatsink design was performed by iterating its dimensions until the desired temperatures were obtained. Figure 2 shows the temperature distribution, and the highest and lowest temperatures registered. It indicates that it is possible to achieve the required differential temperatures. Additionally, the results demonstrate that if the input current is large, the overall temperature of the module, after reaching a minimum value, may rise due to the Joule effect (the current passing through the Peltier pillars generates heat that starts reaching the cold side). Figure 2 presents good results, yet these are for operation in free space, and the next step was to implement a more realistic model.

### 2.3. Full System Modeling

**Figure 3:**
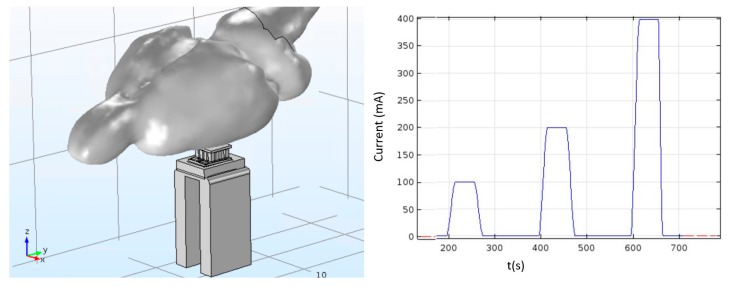
Model that includes the Peltier, heatsink, and rat brain. On the right is the current curve applied to the Peltier over time. The time scale is in seconds and the current levels used were 100 mA, 200 mA, and 400 mA.

After understanding that the Peltier module will be able to reach the desired cold-side temperature in free space, and that it was possible to simultaneously keep the hot side at an acceptable value (the hot side must stay below 43 °C), the next step was to evaluate the performance of the Peltier module while it is being used on a rat brain. Figure 3 shows the developed model, including the rat brain, and the plot of the electric current applied to the device.

The excitation currents were selected taking into account the Peltier datasheet. It shows a range starting at 100 mA and stopping at 1 A. We stopped at 400 mA since that is the maximum value that we can supply with the batteries we have available for our module. Since after applying a current for some time, the cold side temperature reaches a minimum and, depending on the applied current, the cold side may start to heat due to the Joule effect, before applying a new current value, the current is switched off for a few seconds to assure that each simulation starts allays with the same initial conditions.

The properties used for the cooling device’s model were the same as those used for free space operation, and the brain model was built using a material with the characteristics shown in Table 1 [22,41].

With the presence of the new material (brain), the heat transfer in solids module did not work anymore, since the brain has biological mechanisms to control its internal temperature, which must be considered in the simulation. The brain, as a living tissue, has blood vessels that work as a transport for nutrients and oxygen for all the cells, while also regulating our body temperature. If, for some reason, the body temperature goes up, blood flow tends to increase the removal of the excessive heat from the cells. On the opposite side, if the temperature goes down, blood flow is reduced in order to maintain a certain temperature on the cells [22]. This thermal regulation mechanism is important and must be considered in the numerical model of the brain. The bioheat module was used to account for brain’s biological constraints, and to take into consideration the mechanism of temperature regulation. Table 2 shows the parameters that were used to implement the model [41,42,43].

**Figure 4:**
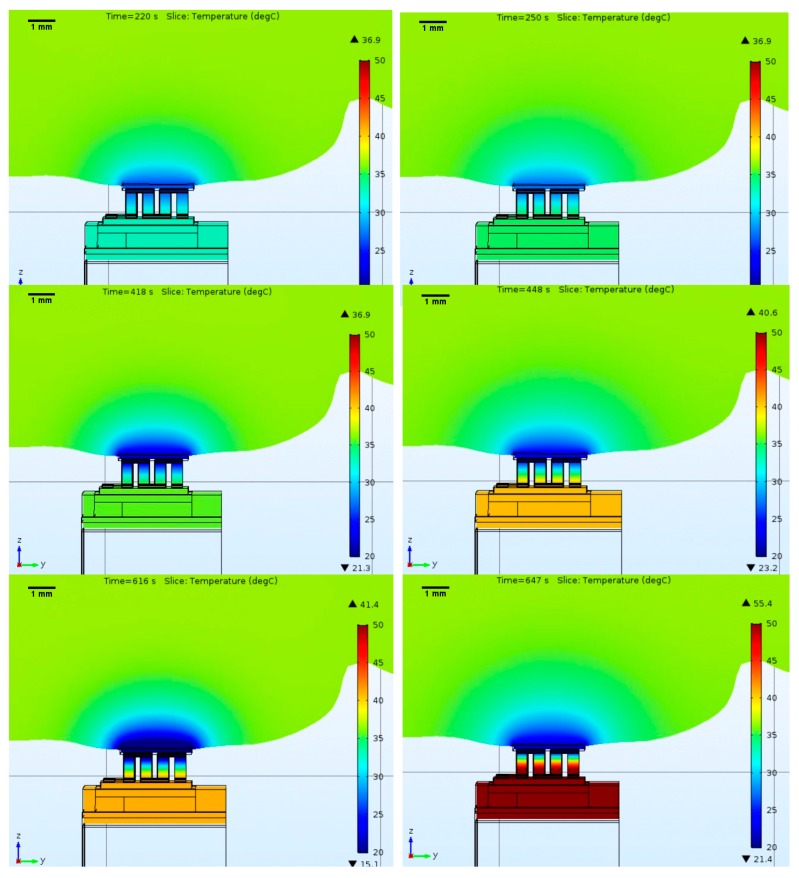
Simulation results with the Peltier module touching the rat brain and the heatsink radiating into air. The figure shows the temperature along the Peltier element, as well as the heatsink region nearby, which will be the hottest area. In addition to the color map showing the temperature distribution, it also shows the ▼-lower, and ▲-higher temperatures present in the structure for the selected time stamps (**top**—100 mA; **middle**—200 mA; **bottom**—400 mA; **left**—minimum temperature achieved; **right**—temperature stabilization).

The simulation started with an initial brain temperature of 37 °C, and a heat sink initial temperature of 24 °C (laboratory temperature), with convection to air at 24 °C (only on the heatsink). Moreover, before turning on the cooling cycle by supplying an electric current, the thermoelectric module was left in the brain for 50 s without any applied current to let the model find an equilibrium point. Figure 4 shows the developed Comsol model.

From previous results, it is possible to observe, as expected, that the brain has a significant effect on the temperatures achieved. Additionally, as expected, the minimum temperatures achieved are not as low as in Figure 2, but it is still possible to get close to 20 °C without going above 43 °C, which is a satisfactory result for the intended application.

## 3. Experimental Performance Assessment

Resorting to simulations, it was possible to verify that the desired temperature is achievable in brain tissue with the proposed Peltier module. The next step was the experimental validation of the numerical data. The cooling device was firstly characterized by resorting to a polystyrene phantom to evaluate its cooling effectiveness. After that, the device was implanted in anesthetized rats and its effectiveness to block neuronal activity was evaluated.

### 3.1. Model Validation

**Figure 5:**
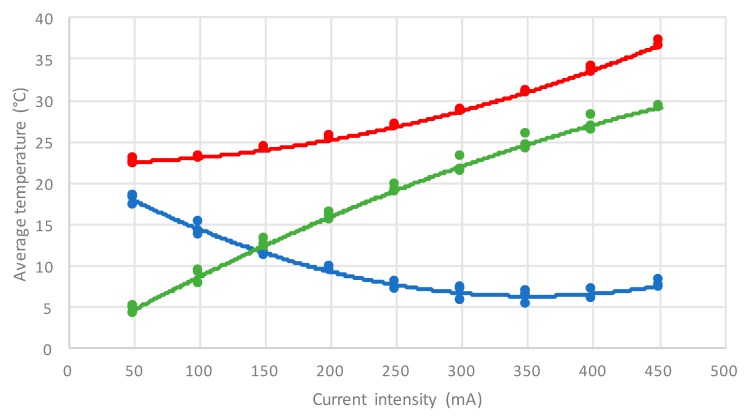
Measurements results obtained on a polystyrene phantom (red line—temperature in the hot side; blue line—temperature in the cold side; green line—temperature difference between hot and cold sides), using a device based on the model of Figure 3, and shown in Figure 6.

**Figure 6:**
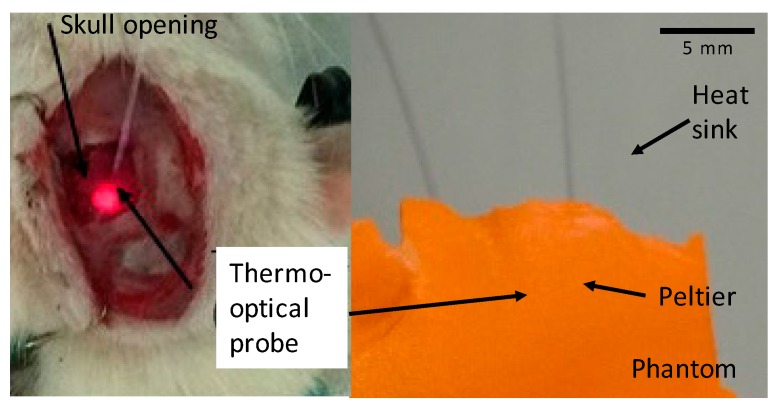
Setup used to perform the experiments and microcooler prototype. Left side shows a detailed rat head opening and temperature probe placement under the Peltier element, and right side shows Peltier testing on a phantom.

Before testing the device in rats, a model verification was made using a phantom ex vivo. A prototype using a Peltier was designed to be placed on top of a polystyrene phantom in order to evaluate the cooling capability. Figure 5 shows the measurement results obtained, with the device shown in Figure 6.

Figure 5 shows that the Peltier implemented in the phantom produces results with values between those of Figure 2 and Figure 4. These results could be due to the fact that the phantom does not implement the brain’s thermal regulation mechanism; therefore, lower temperatures can be achieved with the same applied current.

### 3.2. Thermal Modulation Assessment In Vivo

After the preliminary tests, the microcooler was implanted on the somatosensory cortex of five rats (ipsilateral) to assess its ability to modulate neural activity (Figure 6). The first tests were performed on anesthetized rats, and the modulation was assessed by measuring the sensory response of the cortex to rat vibrissae stimulation. The rat whiskers were stimulated by automated puffs of pressurized air (0.5 Hz) while recording brain activity (local field potentials—LFP) with two copper electrodes positioned on the cortical surface, below the cooling device. Somatosensory evoked potentials (SEP) were recorded with a sampling rate of 15,000 Hz.

**Figure 7:**
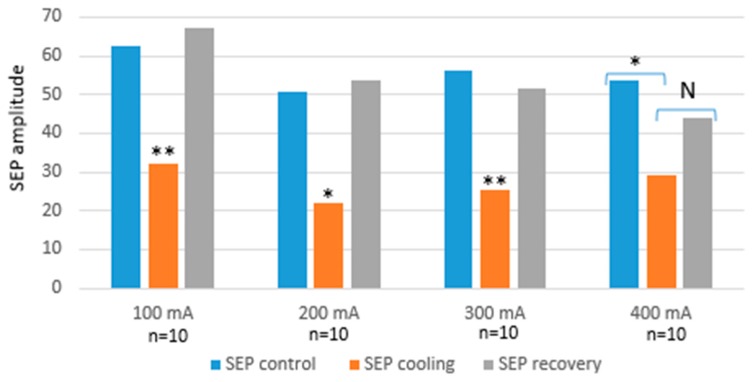
Mean SEP (somatosensory evoked potentials) amplitude in control cooling, and recovery periods. The analysis was based on one-factor ANOVA (amplitude) for *N* = 10 (five rats with two electrodes per rat) for each period for each current (*: *p* < 0.05, **: *p* < 0.01, ***: *p* < 0.001, *N*: no difference).

To evaluate the potential to modulate brain electrical activity, measurements were performed with different current intensities, which started from 100 to 400 mA (100 mA increments). At each intensity, LFP were recorded during three periods: a control period where no current was applied, an experimental period when the microcooler was on, and a recovery period when the device was stopped. We compared the amplitude of the SEP signals between those three experimental conditions. If the cooling is effective, the evoked potentials obtained during the experimental period should be smaller than those from the control and recovery periods. Figure 7 shows the statistical analysis of the measurements performed with two electrodes implanted in five rats.

From the previous figure, it is possible to observe that cooling might have modulated the electrical activity during the stimulation of vibrissae, since the plot “SEP cooling” always shows a reduction in amplitude, compared to the SEP measured during the control period. In addition, no significant difference in SEP amplitude reduction was found between the four intensities tested, maybe because 100 mA was already enough to strongly modulate the neural activity and increasing the current did not add any extra effect. Furthermore, the lack of modulation while increasing the current may also be explained by our simulation data (Figure 4), showing that increasing the current may lead to an extra cooling effect when the Peltier is switched on, but such cooling is followed by a rise in temperature. This is due to the Joule effect from having a high current circulating inside the Peltier, which has a negative contribution on the cold side. A solution for this may be to increase the heatsink’s surface area. Finally, there is no difference in SEP amplitude between the control period and the recovery one. This suggests that the cortex remains functional after the cooling.

## 4. Ambulatory Miniaturized Thermal Modulators

The previous sections allow to conclude about the feasibility of a solid-state thermal neuromodulator that does not require cumbersome accessories to remove the heat produced while cooling a brain tissue volume. We showed that it is possible to reduce brain responses to somatosensory stimulations, suggesting increased cortical inhibition. This is a first encouraging step towards the application of this kind of device for suppressing seizures.

Since the developed model gave some confidence in the ability to predict the thermal performance of such devices, the next step was the design of a miniaturized thermal neuromodulator, so it can be operated without the need of external wiring and be positioned closer to the brain. The next sections will be focused on the analysis of the possibility to obtain a highly-compact device that can be completely implanted on a rat’s skull.

### 4.1. Miniaturized Thermal Modulator

**Figure 8:**
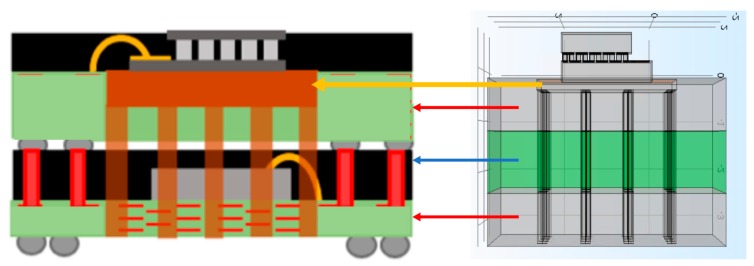
Envisioned miniaturized thermal neuromodulator, and its respective model. The description of elements shown can be found in Figure 1.

The next step towards miniaturization was to select a packaging solution that would enable the integration of the Peltier together with control and communication circuitry, from the future available technologies [44]. Figure 8 shows the proposed miniaturized device, together with the modeling of a fully-working device.

Using the know-how from previous simulations and experiments, COMSOL (COMSOL Group, Stockholm, Sweden) was used to evaluate the most critical issue: the temperature control ability. In this setup, the Micropelt Peltier (Micro MPC-D403) element was considered as the thermal modulator [45]. The biostability of this device is assured since the parts in contact with the brain are either a gold plating (cooler and heat sink) or a medical silicone (the remaining device’s surface).

**Figure 9:**
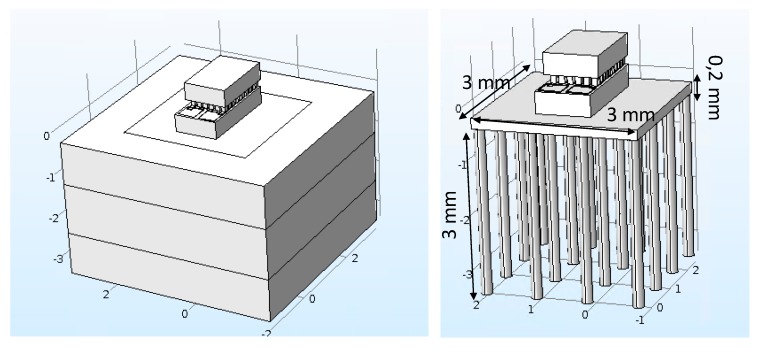
Miniaturized thermal neuromodulator, showing the thermal vias. On the left side, the Peltier is highlighted along with the different packaging levels, and the right side shows the details of thermal vias forming the heatsink.

One main issue with this integration is the heat sink volume reduction. To compensate for that size reduction, thermal vias were considered to transport heat away from the cold side, across the device. Figure 9 shows the device’s model, as well as the details of the thermal vias.

This model was used to assess the device’s performance in different scenarios, as will be described in the next section.

### 4.2. Microcooler Performance Assessment

**Figure 10:**
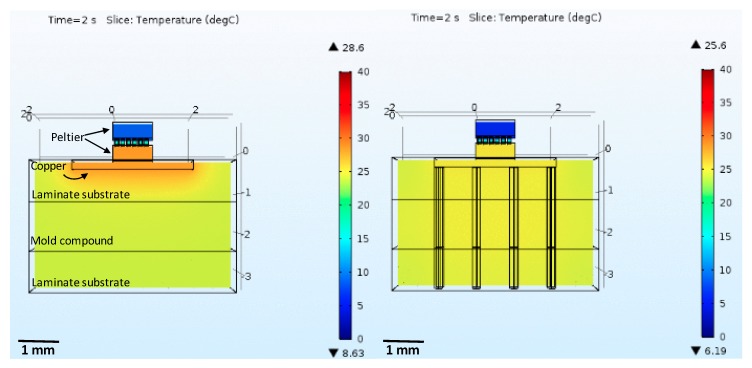
Microcooler performance assessment when operating in free space, with and without thermal vias. The figure shows the temperature along the Peltier element, as well as the heatsink region nearby, which will be the hottest region. In addition to the color map showing the temperature distribution, it is also shows the ▼-lower, and ▲-higher temperatures present in the structure for the selected time stamps.

From a previous work [45], it was concluded that 50 mA would be enough to achieve the temperature required to modulate the neural activity. Thus, it was decided to evaluate the microcooler with that excitation current. Moreover, it was also decided that the air temperature was 24 °C. The results are presented in Figure 10 for two scenarios: with and without thermal vias (copper). The comparison was made at the time stamp when the Peltier cold side temperature reaches the minimum values.

From the previous figures, it is possible to conclude that the thermal vias are effective in removing the heat. Even for this favorable scenario (device surrounded by air), it is possible to observe that the Peltier’s hot side has a lower temperature when the vias are used. Furthermore, it is also possible to observe that the cold side is able to reach temperatures below the 20 °C required for thermal modulation.

**Figure 11:**
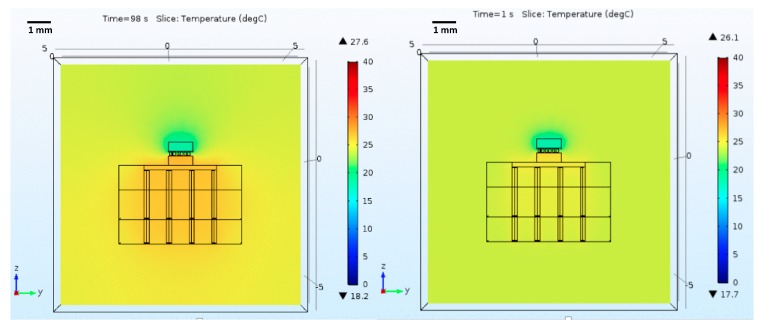
Microcooler performance assessment when operating inside a volume of water. The figure shows the temperature along the Peltier element, as well as the heatsink region nearby, which will be the hottest region. In addition to the color map showing the temperature distribution, it is also shows the ▼-lower, and ▲-higher temperatures present in the structure for the selected time stamps (**left**—minimum temperature achieved; **right**—temperature values for long term operation).

The next simulations considered that the device was placed inside a volume of water at 24 °C. Figure 11 shows the obtained results.

One main conclusion is that the device has a worse performance now than when in free air, but is still able to keep the cold side’s temperature below 20 °C. Additionally, it is possible to observe that for long-term operation (the device is left on until the temperature fully stabilizes), the minimum temperature value does not degrade significantly. Additionally, the performance of the device without thermal vias was assessed, but the hot side’s temperature was too high; therefore, the results were not selected for presentation.

**Figure 12:**
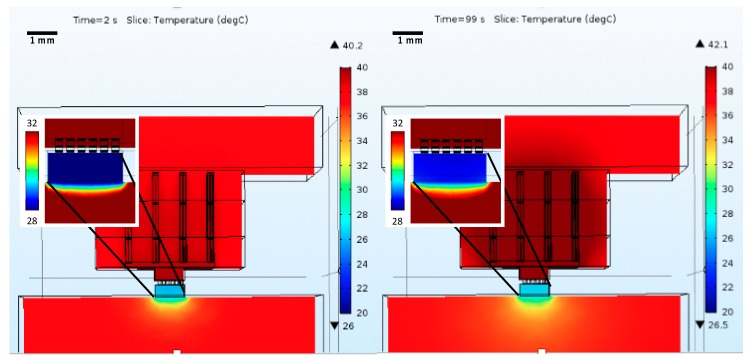
Microcooler performance assessment when operating inside a volume of brain. The figure shows the temperature along the Peltier element, as well as the heatsink region nearby, which will be the hottest region. The inset shows the detail at the Peltier-brain interface, and has a different temperature scale to better display the temperature profile nearby the cooler. In addition to the color map showing the temperature distribution, it is also shows the ▼-lower, and ▲-higher temperatures present in the structure for the selected time stamps (**left**—minimum temperature achieved; **right**—temperature values for long term operation).

Finally, thermal simulations, including the brain phantom, were performed. The model conditions were the same, except for the surrounding environment, which was changed to brain tissue at 37 °C and bone on top. Since the thermal modulator’s complexity was increased, the previously-used realistic brain volume (Figure 3) was replaced by a cube representing the brain’s region of interest, purely for model simplification and since such a large and complex volume was not required. This approximation does not influence the results as the cold volume is well inside the modeled volume. Figure 12 shows the obtained results.

As expected, the minimum temperature value was higher than those from previous scenarios. This is due to two reasons: the brain has a mechanism that attempts to keep the temperature at a certain value, and, also, the brain temperature was set to 37 °C instead of the previous 24 °C. For this scenario, the minimum temperature was not below 20 °C, but still in the 20–30 °C range, which is considered enough to modulate the neural activity according to some literature [5]. One method to decrease the temperature down to 20 °C would be to increase the current. However, that is not desired since the device will consume more power and the long-term results show that the hot side temperature tends to reach values close to 43 °C, the maximum acceptable. To allow for higher current values, the heat sink would have to be modified.

## 5. Conclusions

This paper presents the modeling of a solid-state thermal neuromodulator which can operate without cumbersome systems to properly remove heat. Firstly, numerical modeling allowed the prediction of the achievable thermal boundaries for different scenarios (free space, water, brain model). Thus, based on the modeling, a device was built and tested on a phantom to show that it was effective in obtaining the predicted temperature control. Finally, the device was then used on a rodent model to assess the possibility of promoting thermal neuromodulation.

After verifying that the developed model may be used to predict the Peltier operation, and that a smaller device would be highly desirable from the point of view of energy consumption and thermal management, a new device was proposed. In its current form, such a device may be as small as 5 mm × 5 mm × 3 mm, if the most advanced packaging technologies are used. Despite its small size, this microcooler was able to reduce the temperature to the required values (20–30 °C) to obtain the reduction of neural activity. The simulated temperature decrease of 10 °C (from 37 °C to around 27 °C) reaches depths of up to 1 mm in the brain, with a stable 200 mA supply. Furthermore, with 400 mA, at a 1 mm depth, the temperature can be reduced to about 22 °C, despite the device needing to have a larger heat sink to dissipate all the generated heat.

While the 1 mm depth may not be enough for some applications, it is worth noting that these promising results were achieved without the need to use a cumbersome heat removal scheme, such as pipes with circulating refrigerated fluids.

## Figures and Tables

**Table 1:** Brain tissue model properties.

Brain Property	Value	Reference
Specific Heat—Cp (J/(Kg·K))	3600	[22]
Density—ρ (Kg/m^3^)	1050	[41]
Thermal Conductivity—k (W/(m·K))	0.5	[41]

**Table 2:** Bioheat model parameters.

Bioheat Model Parameter	Value	Reference
Arterial Blood Temperature (ºC)	37	-
Blood Specific Heat—Cp (J/(Kg·K))	3594	[42]
Blood Density—ρ (Kg/m^3^)	1060	[43]
Blood Perfusion Rate (1/s)	0.014	[41]
Metabolic Heat Source (W/s)	16700	[41]
